# Big Tobacco, Alcohol, and Food and NCDs in LMICs: An Inconvenient Truth and Call to Action: Comment on "Addressing NCDs: Challenges From Industry Market Promotion and Interferences"

**DOI:** 10.15171/ijhpm.2019.74

**Published:** 2019-09-15

**Authors:** Peter Delobelle

**Affiliations:** ^1^University of Western Cape, Cape Town, South Africa.; ^2^Chronic Disease Initiative for Africa, Department of Medicine, Faculty of Health Sciences, University of Cape Town, Cape Town, South Africa.

**Keywords:** Big Tobacco, Alcohol, Food, Policy Influence, Non-communicable Disease, LMICs

## Abstract

In their editorial, Tangcharoensathien et al^1^ describe the challenges of industry market promotion and policy interference from Big Tobacco, Alcohol, and Food in addressing non-communicable diseases (NCDs). They provide an overview of the increasing influence of corporate interest in emerging economies and government attempts to implement the World Health Organization (WHO) ‘best buy’ interventions. The authors largely draw on examples from Asia and a few selected countries, but provide little detail as to how aggressive marketing and policy interference plays out in a context of poor legislation and regulation in many low- and middleincome countries (LMICs), where the burden of NCDs is increasing at an alarming rate and governments face a high burden of disease with a limited budget for countering industry interference. This commentary provides some poignant examples of the influence of Big Tobacco, Alcohol, and Food on market regulation and policy interference in LMICs and argues for more policy coherence and accountability in terms of multisectoral action and civil society activism. Securing funds for health promotion and establishing health promotion foundations could help achieve that goal.

## Big Tobacco: Aggressive Marketing and Policy Interference

The tobacco industry has increasingly turned its focus toward emerging markets, seeking for example, to exploit patchwork regulations for tobacco control in sub-Saharan Africa.^2,3^ The high level of tobacco marketing in low- and middle-income countries (LMICs) has been associated with its changing demographics, including a large youth population and rising prosperity,^4^ and with high-income countries (HICs) having more established policies on tobacco control.^5^ For example, whilst being legally challenged in Germany for launching a mass media campaign encouraging smoking among youth in 2011, Philip Morris International (PMI), a leading global tobacco company, continued to roll out its campaign in over 50 countries, including LMICs, some of which suffer the world’s highest rates of tobacco use.^6^

In Indonesia, where tobacco advertising laws are weak, PMI put up massive billboards in streets, and in Brazil, PMI placed its posters at the point of sale, taking advantage of the lack of regulation and inspection to enforce the existing point of sale advertising ban.^6^ PMI was also fined over US$480 000 for violating the National Advertising Self-Regulation Code in São Paulo.^7^ In India, another market with significant opportunity for growth, PMI was criticized for targeting youth,^8^ and in the Philippines PMI promoted their brands across all media.^9^ A study conducted in 2012 among five- and six-year-olds in six LMICs, including Brazil, China, India, Nigeria, Pakistan, and Russia, indicated that 22% of children were able to correctly identify a major PMI brand, including 43% of Chinese youth.^10^

Research conducted in 16 countries found significantly more tobacco advertisements and outlets in LMICs compared with HICs,^5^ and in a study assessing tobacco control in 17 HICs and LMICs that ratified the Framework Convention on Tobacco Control (FCTC), implementation of tobacco control policies was found to be weak, in particular in poorer countries.^11^ Taken together, the evidence shows that tobacco industry strategies aimed at marketing in LMICs abound, and point to the need for comprehensive bans on tobacco advertising, promotion and sponsorship.

Similar tactics are used in Africa, where for example in Guinea, ‘cigarette girls’ are recruited and paid attractive salaries as marketing executives to promote local brands.^2^ In South Africa, where tobacco advertising and sponsorship was banned in January 2001 in line with FCTC recommendations, British American Tobacco (BAT), the largest tobacco company in the world, used viral marketing and young ‘brand amplifiers’ to relaunch its major cigarette brand.^12^ A study of BAT sponsorship in South Africa and Nigeria also reported the use of music as a marketing tool to target young consumers, indicating the limits of national regulatory efforts.^13^ Permissiveness toward tobacco sponsorship in Africa has equally shown to undermine tobacco control support.^14^

In pursuit of its expansion in Africa, BAT has used intimidatory tactics in attempts to suppress health warnings and policy regulation.^15^ As pointed out by Tangcharoensathien et al,^1^ BAT fought courts in Kenya and Uganda in attempts to prevent government from introducing tobacco control measures.^15^ BAT has also been involved in illicit tobacco trade, relying on informal channels to supply markets across Africa since the 1980s, and attempted to gain leverage in negotiations with governments for market access and foreign investment.^16^ Recently BAT has been investigated by the UK Serious Fraud Office on suspicion of corruption over claims that it bribed officials in East Africa to undermine anti-smoking laws,^17^ and accused of avoiding to pay corporate taxes in LMICs, thereby robbing already strained systems of vital resources to pay for healthcare needs inflicted by tobacco-related illness.^18^

In an attempt to shift debate away from the protection of public health, BAT uses corporate social responsibility (CSR) strategies such as supporting tobacco farmers, or funding HIV/AIDS initiatives.^19^ Internal BAT documents revealed how similar tactics were used in China, where illicit trade was a critical factor in its market expansion,^20^ and where CSR was used to secure access to policy-makers.^19^ As in HICs elsewhere, CSR as a political activity has been instrumental to protecting Big Tobacco in LMICs, in combination with lobbying and the offer of voluntary self-regulation codes,^4,21^ which the industry frequently complements with ineffective mass education programs. In the early 1990s, for example, PMI launched youth smoking prevention programs in Latin America, enabling them to undermine tobacco control interventions required by the FCTC.^22^

## Big Alcohol: The Illusion of Responsibility

Like Big Tobacco, the global alcohol industry has expanded its market in LMICs in the last decades. Diageo and SABMiller, two of the largest alcohol industries, reformed their corporate structure and operations to maximise opportunities for expansion across LMICs.^23^ In spite of the evidence that alcohol policies, in particular those related to price and availability, and advertising bans are effective strategies to reduce alcohol-related harm and are associated with lower alcohol consumption in LMICs,^24,25^ the alcohol industry and its front groups actively promote partnership with governments to influence national alcohol policies,^26^ eg, through the International Center on Alcohol Policies, which was co-founded by PMI.^27^ National alcohol policy documents in Lesotho, Malawi, Uganda, and Botswana for example were all found to reflect industry interests, focusing on individual behaviour and ignoring more effective population based interventions.^26^

In India, strategies for expansion by Diageo in 2013 and 2014 focused on India’s younger generation, women, and the emerging middle class for growth opportunities.^28^ Questionable practices are used, for example by Heineken, which employs ‘beer girls’ in Africa and Asia, who are hired by bar owners to circulate on the premises and encourage men to drink more.^29^ Although the practice has existed for a long time and Heineken repeatedly promised to abandon the strategy, it only made headlines in 2018 when the Global Fund to Fight AIDS, TB, and Malaria suspended its planned partnership with the alcohol giant because of “the company’s use of female beer promoters in ways that expose them to sexual exploitation and health risks.” ^30^

Investigation also showed how Heineken collaborates with dictators and authoritarian governments, avoids taxes, and has been linked to human rights violations and high level corruption.^29^ CSR initiatives, used to influence the framing of alcohol-related issues in line with industry interests, but not shown to reduce harmful drinking,^31,32^ include eg, the introduction of a successful workplace program for voluntary HIV counselling, testing and treatment in Rwanda,^33^ and support of an annual symposium to create awareness of ‘the health and nutritional benefits of beer consumption’ in Nigeria.^34^ In South Africa, SAB in 2017 initiated a campaign targeting university students as beneficiaries of a food supplementation project funded by proceeds of beer sales, but it suspended the program after significant pressure from the public health community.^35^

In South Africa, the alcohol industry also established an Association for Responsible Alcohol Use as a front group and seeks to influence government policies by eg, sponsoring trips for parliamentarians, funding educational workshops, and partnering with the Department of Trade & Industry to sponsor underage drinking initiatives.^36^ In doing so, the alcohol industry tries to obtain a legitimate platform for lobbying against proposals around the reduction of alcohol availability, taxation, and marketing restrictions, despite the global evidence showing that these are cost-effective measures to decrease alcohol-related harm.^36,37^ In an effort to reduce alcohol consumption, in 2018, a Liquor Amendment Bill was introduced to South Africa’s Parliament, proposing a ban on all alcohol advertising, but this met with strong opposition from the industry.^38,39^

## Big Food and Soda: Another Game of Thrones

Similarly, the food industry interferes with policy in LMICs through lobbying and securing buy-in from policy-makers, as illustrated for example by the influence of the soda industry on obesity science and health policy in China.^40,41^ Coca-Cola, the world’s largest producer of sugar-sweetened beverages (SSB), also funds scientists who shift the blame for obesity away from bad diets, towards promoting a balanced and active lifestyle,^42,43^ despite strong evidence of the impact of an SSB tax.^44^ Increased levels of SSB consumption have been blamed for fuelling the obesity epidemic worldwide, which in LMICs adds to an already high burden of undernutrition and infectious diseases.^45^ A recent study which showed that globally, children are exposed to high volumes of television advertising for unhealthy foods and beverages, adds more fuel to the fire.^46^

The food industry has also rapidly expanded in LMICs, as a result of mass-marketing campaigns and foreign investment, primarily through takeovers of domestic food companies.^47^ In China and India, Coca-Cola successfully re-entered the market after liberalization of their economies, and sought to influence government and key organizations to increase consumption and challenge public health policy.^48^ Big Food is a driving force behind the rise in SSBs consumption and processed foods rich in salt, sugar and fat,^49^ which has been linked to the rising levels of obesity and diabetes in LMICs.^50^ In South Africa, the number of transactions from fast food chains has increased significantly in the last decades, many of which are linked to soft drink companies, and food manufacturers have increased their market shares by making their foods more available, affordable, and acceptable.^51^

Like the tobacco and alcohol industries, major food manufacturers and retailers in South Africa have active CSR programs, with an often-strong focus on nutrition education. The food industry has taken voluntary action on food marketing through the South Africa Pledge on Marketing to Children, but there are many exclusions related to the packaging and labelling, and so far none of the signatories, including food manufacturers, retailers, and fast food chains, have made any real commitments.^51,52^ In a study assessing the content of television food advertisements in South Africa, nearly half of the ads viewed during child and family time were for food, including desserts and sweets, fast foods and sweetened drinks.^53^ There were also three times as many ads for unhealthy foods and beverages as for healthy foods during peak times, despite government regulation and the industry code for self-regulation applied through the Advertising Standards Authority of South Africa.^46^

So far, the changes in South Africa’s food industry practices have contributed to a steady increase in per capita food supply of fat, protein, and calories, which appear to be associated with unhealthful changes in dietary patterns and increased sales in most categories of packaged foods.^51^ South Africa acts as a trade hub for the Southern African Development Community (SADC),^54^ and several studies have documented an increased consumption of processed foods and sugary beverages in all SADC countries since the mid-1990s, along with decreased consumption of healthier traditional foods.^55,56^ Processed and packaged foods and beverages are also rapidly reaching food insecure populations in other LMICs, as shown for example when looking at consumption patterns in Mexico and China, and pose a major challenge to the food system and the health of poor and vulnerable populations.^57^

## Discussion

Reducing the impact of tobacco, unhealthy diets and harmful alcohol consumption worldwide, and in LMICs in particular, constitutes a grand challenge for the public health community, and requires understanding of how these ‘industrial epidemics’ contribute to the rising burden of non-communicable diseases (NCDs) through the promotion of products damaging to health and indirectly through influence over public policy.^58^

As this commentary has shown, Big Tobacco, Alcohol, and Food use opportunistic strategies to enter LMICs, where industry marketing and policy interference is less controlled and monitored. The continued profitability of these industries is linked to their ability to resist policy regulation, but also to the speed with which they exploit the relative lack of regulation in LMICs and switch their focus away from the traditional markets.^18^ As regards Big Tobacco, WHO has actively sought to monitor and contain industry influence, with FCTC Article 5.3 requiring to protect public health policies ‘from commercial and other vested interests of the tobacco industry.’ Its approach to food and alcohol industries, however, is different, with both its Global Strategy on Diet, Physical Activity and Health^59^ and more recent Global Strategy to Reduce the Harmful Use of Alcohol^60^ assuming scope for partnership and cooperation, which the FCTC precludes.^58^

The active promotion of international interests of the alcohol industry contrasts with attitudes towards the interest of tobacco companies. Given that the harm caused by alcohol is much greater in LMICs and that alcohol use has become the single largest behavioural risk factor for disease and disability in middle-income countries,^61^ concerted policy actions are required to reduce alcohol consumption in LMICs.^62^ Alcohol is subject to less stringent forms of regulation, and the alcohol industry continues to play a role in policy-making in many countries worldwide.^63^ Given the many similarities that exist, however, between the tobacco and alcohol industries in terms of marketing and political strategies, there is a clear need for policy coherence and monitoring of policies that regulate alcohol marketing and availability, in particular in LMICs.

Finally, as regards the global and increasing impact of the food industry on the rising burden of NCDs and childhood obesity, in LMICs in particular, a stricter monitoring of policy is warranted. Implementation of evidence-informed policies, and monitoring of industry driven policy interference is paramount to achieving this objective and will require continued attention of health policy-makers, civil society and the public health community. Setting up a framework legislation for NCDs extending beyond tobacco control may be a good option to help achieve this goal, including Sustainable Development Goal (SDG) 3.5, which aims at strengthening the prevention and treatment of substance abuse, including the harmful use of alcohol. Given that Big Tobacco, Alcohol, and Food use similar strategies to undermine NCD prevention and control, policies aimed at regulating and preventing the harm caused by these industries should hence be based on policy coherence. Although not a substitute for detailed legislation governing tobacco, alcohol, and food marketing and control, it could have the potential to accelerate national progress by raising the political profile of NCDs, and clarifying who is accountable for coordinating a cross-sectoral response.^64^

In addition to paying more attention to broader governance and regulatory structures to identify and reduce industry interference on NCD prevention laws and policies,^65^ it is however also necessary to examine the role of civil society. Examples of best practices and challenges faced by civil society acting against transnational corporations in a neoliberal policy environment exist and have been documented.^66^ Advocacy and activism responding to products, practices or policy influence from the Big industries can mediate negative health impacts, which in turn can be supported by securing funds for health promotion, such as adopting a policy earmarking ‘sin’ taxes for the creation of health promotion foundations.^67-69^ Activism is also needed to hold governments accountable for national NCD responses and implementing whole-of-government and -society approaches, underpinned by multisectoral action for health.^70^ In South Africa, for example, government and civil society have since the introduction of democracy in 1994 used regulation, public education, health services, and community mobilization to address corporate practices that increase NCD risk.^54^ As highlighted in the Montevideo Roadmap 2018-2030 on NCDs, firm commitment and support from the international community is however needed if the goal of reducing by 2030 by one third premature mortality from NCDs (SDG 3.4) in LMICs is to be achieved.

## Acknowledgements

In remembrance of Prof Emeritus David Sanders, global public health pioneer and advocate of people’s health, who recently passed away whilst on holiday. His invaluable contributions to advancing primary healthcare based on the principles of social justice and equity and his fierce criticism of the impact of neoliberalism on global health will forever be remembered by his colleagues and friends in the public health community and beyond.

## Ethical issues

Not applicable.

## Competing interests

Author declares that he has no competing interests.

## Author’s contribution

PD is the single author of the paper.
